# Ulcer over Chin in an Immunocompromised Individual

**DOI:** 10.4269/ajtmh.2010.09-0667

**Published:** 2010-06

**Authors:** Dincy Peter, Meera Thomas, Mary Mathews

**Affiliations:** Departments of Dermatology, Pathology, and Microbiology, Christian Medical College, Vellore - 632004, Tamil Nadu, India

**Figure 1.**
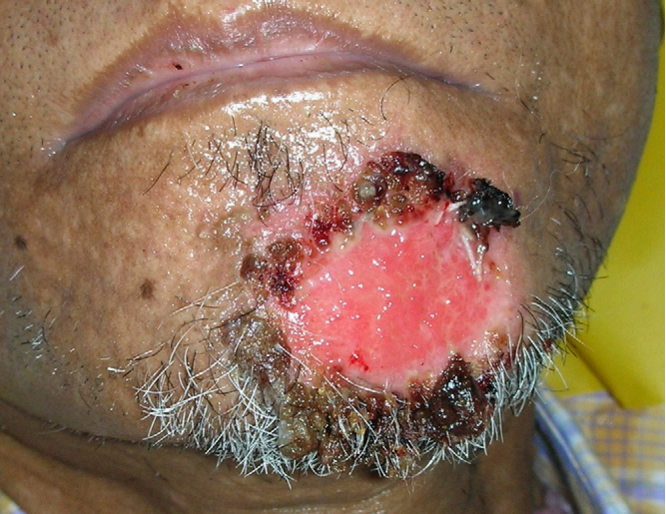
Superficial ulcer with adherent crusts over the chin. This figure appears in color at www.ajtmh.org.

**Figure 2.**
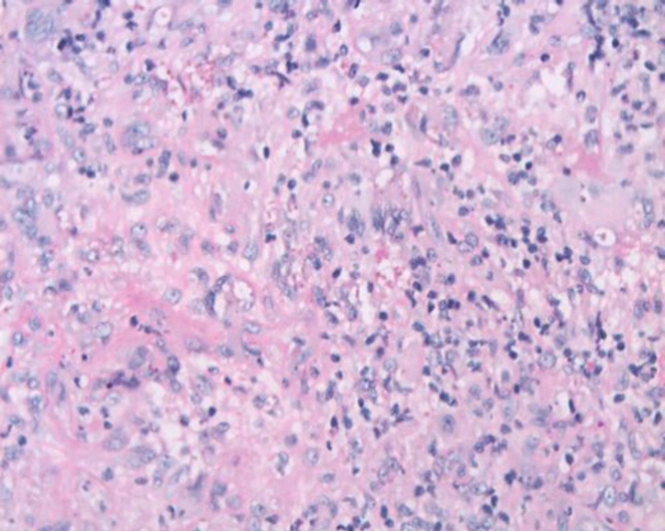
Yeast and sausage shaped structure with central septation, PASD 400×. This figure appears in color at www.ajtmh.org.

**Figure 3.**
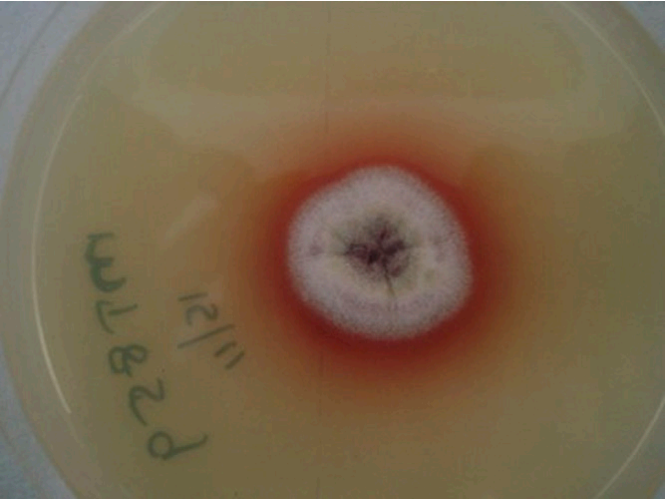
Greenish yellow, flat, velvety colony with surrounding diffusible red pigmentation. This figure appears in color at www.ajtmh.org.

A 45-year-old human immunodeficiency virus (HIV)-positive male from northeastern India presented with fever and cough as well as a 3-month-old ulcer over the chin. On examination, there was a 5 × 5-cm superficial ulcer with a thick adherent crust over the chin (Figure 1). The individual had three papules with central umbilication over the neck and face. He had anemia (hemoglobin = 7.5 g) and a cluster of differentiation 4 (CD4) count of 89 cells/μL. His chest radiograph and computed tomography (CT) scan of the thorax showed right mid- and lower-zone consolidation. Histopathology of the umbilicated papule and the ulcer showed numerous yeast forms and a few sausage-shaped structures with central septation within the histiocytes (Figure 2). Fungal tissue cultures from skin lesions and bronchoalveolar lavage aspirates showed a greenish yellow, flat, velvety wrinkled colony with surrounding diffusible red pigment (Figure 3). This was diagnostic of *Penicillium marneffei* infection. Sputum smears and culture were negative for tuberculosis. Direct immunofluorescence detection for *Pneumocystis carinii* from sputum sample was negative.

Mucocutaneous lesions are seen in two-thirds of patients with *Penicillium marneffei* infection. These include generalized papular eruptions, central umbilicated papules resembling molluscum contagiosum, acne-like lesions, folliculitis, subcutaneous nodules, necrotic nodules, papules and nodules with ulceration, oropharyngeal papules, palatal perforation, and genital papules.^1^
